# Measurement instruments for quantifying physical resilience in aging: a scoping review protocol

**DOI:** 10.1186/s13643-019-0950-7

**Published:** 2019-01-28

**Authors:** Sue Peters, Theodore D. Cosco, Dawn C. Mackey, Gurkaran S. Sarohia, Jeffrey Leong, Andrew Wister

**Affiliations:** 1 Gerontology Research Center, Simon Fraser University, 2800-515 West Hastings St., Vancouver, V6B 5K3 Canada; 2 Department of Physical Therapy, Faculty of Medicine, University of British Columbia, 212 - 2177 Wesbrook Mall, Vancouver, British Columbia V6T 1Z3 Canada; 3 Oxford Institute of Population Ageing, University of Oxford, 66 Banbury Road, Oxford, OX2 6PR UK; 4 Department of Biomedical Physiology and Kinesiology, Simon Fraser University, 8888 University Drive, Burnaby, British Columbia V5A 1S6 Canada; 5 Centre for Hip Health and Mobility, 766-2635 Laurel Street, Vancouver, British Columbia V5Z 1M9 Canada; 6 MD Undergraduate Program, Faculty of Medicine, University of British Columbia, 317-2194 Health Sciences Mall, Vancouver, British Columbia V6T 1Z3 Canada; 7 Department of Gerontology, Simon Fraser University, 2800-515 West Hastings St, Vancouver, British Columbia V6B 5K3 Canada

**Keywords:** Resilience, Scoping review, Senior, Function, Mobility, Physical

## Abstract

**Background:**

Physical resilience is the ability to optimize or recover motor function in the face of disease, injury, or aging-related decline. Greater knowledge of how some individuals regain or maintain function despite pathology may help identify protective factors and approaches that promote healthy aging. To date, a scoping review on physical resilience has not been conducted. The aims are to (1) identify measurement instruments for physical resilience, (2) synthesize and map the key concepts of physical resilience, and (3) identify gaps and make recommendations for future research.

**Methods:**

A scoping review of Scopus, Web of Science, Cumulative Index to Nursing and Allied Health Literature, Medline Ovid, PsycINFO, and AgeLine databases will take place using the search strategy “resilience” AND (aging OR elderly OR older adult). The initial electronic search will be supplemented by hand searching the reference lists and review articles to identify any missing studies. Two parallel independent assessments of study eligibility will be conducted for the title, abstract, and full-text screens. To meet study inclusion criteria, the term “resilience” must be applied in relation to the physical health of older adults. Any disagreement will be resolved by consensus and a third reviewer consulted to make a decision if consensus is not achieved initially. Physical resilience information to be extracted are measurement instruments that describe the core domains of (1) body function or structure (signs or symptoms, etc.), (2) activity and participation (quality of life, etc.), and (3) societal impact. Tables and/or charts will map the data with distribution of studies by core domains. Finally, the amalgamation of results will be an iterative process whereby reviewers will refine the plan for presenting results after data extraction is completed so that all of the contents of the extraction may be included in the results.

**Discussion:**

The information gleaned in this scoping review will be essential to understand how physical resilience is currently measured and identify gaps for further research.

**Electronic supplementary material:**

The online version of this article (10.1186/s13643-019-0950-7) contains supplementary material, which is available to authorized users.

## Introduction

Resilience is the ability to resist or recover from the adverse effects of a stressor [1–3]. It is an active, adaptive process and not merely the absence of disease or pathology [4]. In recent years, resilience approaches have been applied to older adults challenged by mental health [5–7]. Furthermore, the complex construct of resilience is attributed to adjustment to the aging process [3]. Often accompanying aging, however, is the onset of acute and chronic illnesses or diseases [8, 9]. The impact of these conditions broadly affects many aspects of life for older adults, such as activities of daily living, social roles, and mental health [10]. Commonly, aging literature has focused on the pathogenic aspects of illness or disease [11] with recent growing interest in the more positive aspects of aging, namely resilience [12–14]. Considering that the global population is aging [11, 15], the quality of these extended years has become of great interest. Further, models of resilience are being favored over successful aging models [16]. Compared with illness or disease, resilience is greatly understudied, and yet, it is a significant explanatory variable for successful aging [12, 17–19].

Complicating our understanding of aging within illness or disease is that multiple trajectories of physical recovery are observed and cannot be explained by biological factors alone [20–22]. An emerging concept stemming from this work is “physical resilience,” which is the ability to recover or optimize function in the face of diseases or age-related losses [2, 23]. A working definition of physical resilience for older adults conceptualizes it “at the whole person level: a characteristic which determines one’s ability to resist or recover from functional decline following health stressor(s)” [2]. Considering the challenges older adults face to maintain motor function with aging [24, 25], a high level of physical resilience may increase the number of years of independent community mobility and reduce an individual’s injury and mortality risk.

Beyond impairment to motor function, physiological stressors can affect physical resilience. In a National Institute on Aging’s report to identify measures of physiologic resiliencies in aging, multiple types of resiliency outcomes were identified that included functional and physiological processes [1]. Heart rate, blood pressure, postural sway, and other measures were suggested as physiological responses to physical stressors [1]. Furthermore, physical resilience contains a variety of phenotypes, with many physically resilient individuals differentiated by neuroendocrine or genetic variations associated with resilient trajectories [4]. Critically, few reports examine connections between physiological measures and physical motor outcomes such as return to independent community mobility.

A review of methodological approaches to operationalize resilience found three main methodologies for resilience measurement were psychometric, definition-driven, and data-driven approaches [26]. Hence, quantifying physical resilience may employ a diversity of measurement instruments; it is likely that a large body of literature needs to be examined for a more complete framework of how physical resilience is measured to capture the personal (including biological) and psychosocial resources supporting recovery [27].

Whitson et al.’s review and Hadley et al.’s report examined aspects of physical resilience, namely definitions and physiological parameters; however, these reviews examined two databases and did not pull together physiological and motor function parameters in one scoping review [1, 2]. Boers et al. describe a comprehensive conceptual framework of core areas for outcome measurement for rheumatology and may be a useful template for other areas of health, such as an examination of physical resilience in aging [28]. We will expand upon these reviews by broadening the spectrum of included studies with a wider search strategy and additional databases. A wide search strategy is necessary to uncover potential connections between physiological and motor function parameters, as well as outline prospective biopsychosocial factors that may foster physical resilience. Thus, the objectives of the proposed review are to: (1) identify measurement instruments for physical resilience based on Boers et al. [28], (2) synthesize and map the key concepts of physical resilience, and (3) identify gaps and make recommendations for future research. This paper outlines the protocol and the details of these key objectives for this scoping review.

## Methods

### Study design

**Fig. 1:**
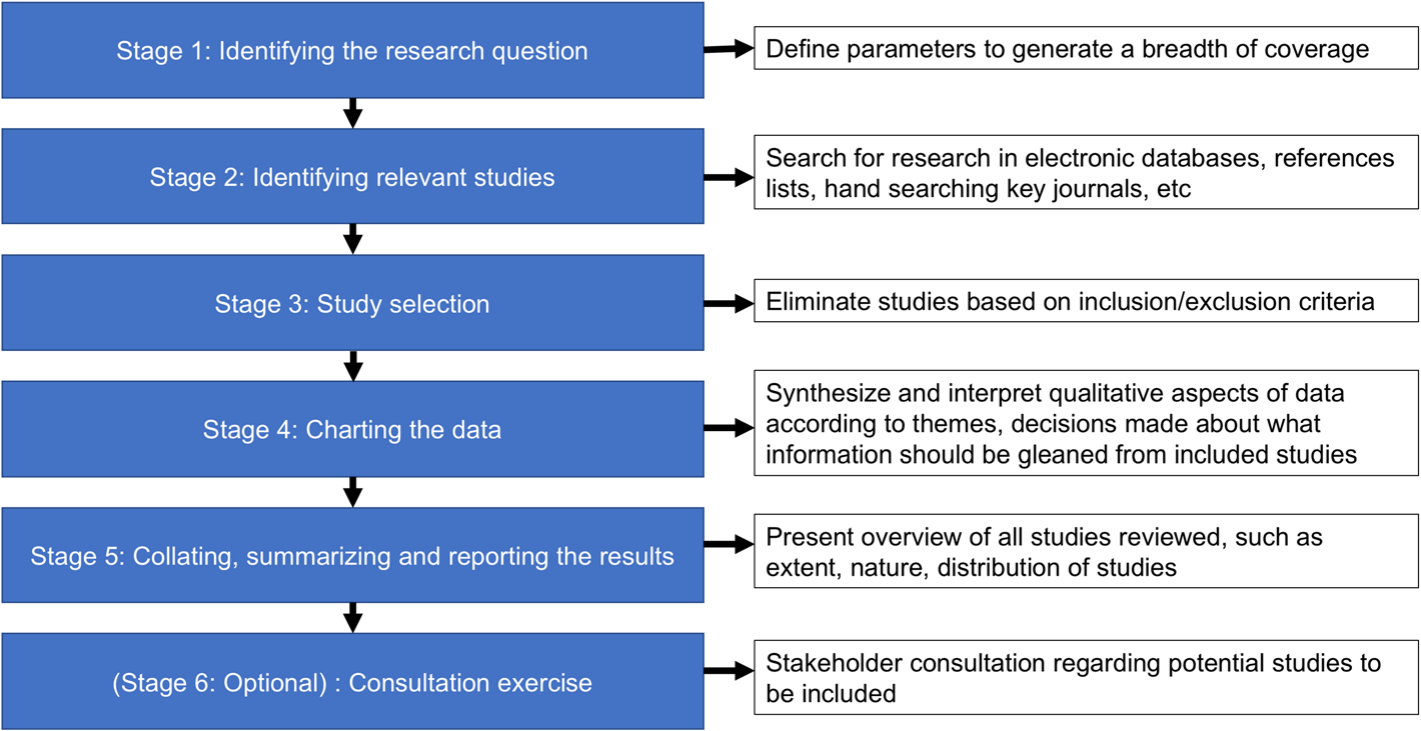
Schematic of Arksey and O’Malley’s [29] Six Stage Framework for Scoping Reviews

To discover measurement instruments and prospective links among physiological and motor function factors, a scoping review search strategy is necessary. The methodology for this scoping review is guided by Peters et al., Tricco et al., Levac et al., and Arksey and O’Malley [29–32]. The stages of this scoping review will follow Arksey and O’Malley proposed guidelines and are outlined in schematic form in Fig. 1 [29]. A populated PRISMA-P checklist in this study protocol can be found in an additional file [see Additional file 1].

### Search methods for study identification

We will conduct a search from inception to March 14, 2018, that will initially be applied in MEDLINE Ovid and then run in the following electronic databases: Scopus [33], Cumulative Index to Nursing and Allied Health Literature (CINAHL), MEDLINE Ovid, Web of Science, PsycINFO, and AgeLine. To maximize the sensitivity of the search, both Medical Subject Headings (MeSH) and keyword searches will be used. The following MeSH terms will be used: “resilience, psychological,” “aging,” and “aged,” with keyword searches of terms such as “resilience,” “aging,” “ageing,” “elderly,” and “older adult,” based on the search terms used in previous resilience reviews [26, 34]. If a database does not index citations using MeSH terms, they will not be used, and only keywords will be used. The initial electronic search will be supplemented by hand searching the reference lists from included studies to identify any missing studies. Additionally, previous reviews will be identified on this topic and their reference lists searched to ascertain whether potentially eligible studies were missed. Additional file 2 shows the search strategy for all electronic databases as well as the platform or interface used to access the database.

### Selection of studies

We will upload search results into EndNote (x6.0.2 Thomson Reuters). Prior to screening, reviewers will undergo training to ensure basic understanding of the background of the field and purpose of the review. After duplicates are removed, abstracts and titles will be screened for inclusion by two independent reviewers. Calibration with a subset of approximately 50 title and abstract records will take place early in the review process to ensure comprehension of the eligibility criteria and consistency of rating between all reviewers. This step will ensure early correction of any systematic patterns of discrepancies that may arise between reviewers and determine whether the instructions for screening are sufficient. Next, eligibility will be assessed with full-text screening by two independent reviewers. If the relevance of an abstract is unclear, it will be reviewed with full-text screening [29]. A second round of calibration between reviewers will occur at this stage during full-text screening to ensure consistent flagging of full-text articles for data extraction. Any disagreement will be resolved by consensus and a third reviewer consulted to make a decision if consensus is not achieved initially.

### Inclusion criteria

We will include studies of older adults (≥ 65 years old). To be included, the term “resilience” must be applied in relation to the physical health of older adults.

### Exclusion criteria

Studies will be excluded if: (1) no physical resilience or motor/physiological function is measured; (2) ineligible article (e.g., conference proceedings, editorial); (3) beyond the individual (e.g., climate, war); (4) resilience characterized as a psychological parameter (e.g., mental health) or personality trait; (5) resilience of soil, oil, ecosystem, pipelines, animals, bugs, computers/devices, etc.; (6) brain resilience without motor function, such as cognitive resilience or reserve; (7) organ, molecular, cellular, metabolism, and genetic resilience without links to physical health; and (8) non-English language article.

### Data extraction

Boers et al. suggests that their comprehensive conceptual framework for outcome measurement developed for a rheumatology patient population may be a useful template for other areas of health care [28]. We are primarily interested in measurement instruments that measure physical resilience in one or more of the following domains of the International Classification of Functioning, Disability, and Health (ICF) [35] in older adults with pathology. These include: (1) body function or structure (signs or symptoms, biomarkers, etc.), (2) activity and participation (quality of life, etc.), and (3) societal impact of the pathology [28]. Specifically, we will include any domains that elucidate patient-centered, intervention-specific, or cross-sectional information that describe, in relation to physical resilience, the core areas of: (1) death, (2) life, (3) resource use or economic impact, and (4) pathophysiologic manifestations [28].

Secondary measurement instruments will be considered, depending on what is uncovered during the review; anticipated possibilities include heart rate, blood pressure, postural sway, neuroendocrine, genetic, blood/urine or brain measures, and other physiologic measures that directly relate to motor outcomes. To support the potential breadth of physical resilience research, all study designs will be included as long as the inclusion criteria are met.

Two reviewers will independently extract the following data: (1) author(s); (2) year of publication; (3) source/country of origin; (4) study population, sample size, participant characteristics such as injury or pathology; (5) study methodology (e.g., longitudinal, cross-sectional, experimental); (6) outcome domains (e.g., death, life, resource use, pathophysiological); (7) measurement instruments to quantify domains pertinent to physical resilience; and (8) any other key findings related to physical resilience not captured by the previous variables that may be classified as supplementary secondary measurement instruments (described below). If data in an included study are unclear or missing, the authors will be contacted for clarification. Discrepancies between reviewers for the extracted data will be resolved with discussion and involve a third reviewer if consensus is not achieved initially. To guide the conceivable scenario where multiple measurement instruments are used to quantify physical resilience for a given study, all measurement instruments that are highly validated (e.g., using published validity data) will be extracted, such as the Connor Davidson Resilience Scale with acceptable levels of internal consistency, convergent/discriminant validity, and theoretical construct validity [34]. Measurement instruments *without* published validity or reliability data will be extracted and noted as such. Validity or reliability of the measurement instruments will be verified with a literature search.

### Data synthesis and gap identification

The extracted data will first be used to generate a literature overview with a quantitative and qualitative analysis following, in a parallel and integrative manner [36]. The quantitative or numerical analysis will compile descriptive statistics of the extent, nature, and distribution of studies included in the review including pathology, outcome domains, and measurement instruments. We will differentiate between primary and secondary outcomes with tables and/or charts that will map the data with distribution of studies by study population, outcome domains, and measurement instruments. Classification will be based on consensus among the authors.

The qualitative analysis will involve synthesizing the evidence against our research objectives using a narrative “charting” approach [29, 32]. We consider charting to be a process by which qualitative information is synthesized and interpreted by sorting and sifting material into key themes and issues [29]. The qualitative synthesis of the evidence will identify research gaps based on the “Conceptual Framework of Core Areas for Outcome Measurement,” suggested by Boers et al. (2014) with concepts (e.g., health condition impact, pathophysiology), core areas (e.g., death, life impact, resource use, pathophysiology), and domains (e.g., the International Classification of Functioning, Disability, and Health) [28]. Gap identification will detect areas, such as patient populations or countries in the world, that lack research on physical resilience. Importantly, since this scoping review will not include an evaluation of the quality of the identified studies, the data synthesis will not necessarily identify gaps where research is of poor quality. Gaps will be rank ordered from highest to lowest priority for future research, with priority level determined by areas with the least research given the highest priority.

Measurement instruments that may not directly apply the term “resilience” to health in older adults but have potential for such use will be considered as supplementary measurement instruments founded on what the scoping review process discovers. These supplementary measurement instruments will be summarized and categorized as potential measures for further study. If initial disagreement is present between two reviewers regarding measurement instruments, consensus will be attained between two reviewers with a third reviewer consulted if agreement is not achieved initially. Finally, amalgamation of results will be an iterative process whereby reviewers will refine the plan for presenting results after data extraction is completed so that all of the content areas will be included. Based on Arksey and O’Malley’s recommendations, we will elect to omit the optional consultation stage [29, 37].

## Discussion

This scoping review will be the first to summarize measurement instruments for quantifying physical resilience in aging. We anticipate that the results of this research may highlight important physiological biomarkers, motor function measures, and potentially modifiable factors. This information will be essential to mapping how physical resilience is currently measured and identify gaps for further research. As physical resilience is a concept with emerging evidence, a scoping review is the best approach to ensure the broadest possible data be extracted and analyzed. Understanding how these potential biopsychosocial factors contribute to recovery may outline ways that resiliency could be fostered in the rehabilitation process. Fostering physical resilience may improve pre-habilitation or preventative strategies to promote recovery, and could improve acute to chronic care management.

## Additional files

Additional file 1: PRISMA-P checklist. This file (.docx) contains the completed PRISMA-P checklist. (DOCX 33 kb)

Additional file 2: Search strategy for MEDLINE Ovid. This file (.docx) contains the search strategy for MEDLINE Ovid. (DOCX 14 kb)

## Abbreviations

CINAHL: Cumulative Index to Nursing and Allied Health Literature
ICF: International Classification of Functioning, Disability, and Health
MCID: Minimal clinically important difference
MeSH: Medical Subject Headings
NIA: National Institute on Aging
