# Supplementary material for: Measurement instruments for quantifying physical resilience in aging: a scoping review protocol
Source: Syst Rev. 2019 Jan 28;8:34. doi: 10.1186/s13643-019-0950-7 (PMC6348652; doi:10.1186/s13643-019-0950-7)
Supplement: Supplementary file 2 — Search strategy for MEDLINE Ovid. This file (.docx) contains the search strategy for MEDLINE Ovid. (DOCX 14 kb) [file 13643_2019_950_MOESM2_ESM.docx]

Search Strategies

MEDLINE (through Ovid platform) = 4340 results

| *1. RESILIENCE, PSYCHOLOGICAL/* |  |
| --- | --- |
| 2. resilien*.mp. |  |
| 3. *Aging/* |  |
| 4. *Aged/* |  |
| 5. older adult*.mp. |  |
| 6. elderly.mp. |  |
| 7. ageing.mp. aged.mp |  |
| 8. 1 or 2 |  |
| 9. 3 or 4 or 5 or 6 or 7 |  |
| 10. 8 and 9 |  |

CINAHL (through EBSCO platform)

(aging OR aged OR older adult* OR elderly OR ageing) AND (resilien*) = 1600 results

PsycINFO (through EBSCO platform)

(resilien*) AND (aging OR aged OR older adult* OR elderly OR ageing) = 3,956 results

AGEline (through EBSCO platform)

(resilien*) AND (aging OR aged OR older adult* OR elderly OR ageing) = 736 results

Scopus
(resilien* OR *RESILIENCE, PSYCHOLOGICAL/*) AND (*Aging/* OR *Aged/* OR older adult* OR elderly OR ageing) = 6914 results

Web of science

(resilien*) AND (aging OR aged OR older adult* OR elderly OR ageing) = 4613 results

Legend of MESH terms:

*RESILIENCE, PSYCHOLOGICAL/*

*Aging/*

*Aged/*

Legend of keywords:

aging

aged

older adult

elderly

ageing
